# Evaluating the perceived affective qualities of urban soundscapes through audiovisual experiments

**DOI:** 10.1371/journal.pone.0306261

**Published:** 2024-09-05

**Authors:** Maria Luiza de Ulhôa Carvalho, Margret Sibylle Engel, Bruno M. Fazenda, William J. Davies

**Affiliations:** 1 Faculty of Visual Arts, Federal University of Goiás, Goiânia, Brazil; 2 Chair of Acoustics and Haptics, Technische Universität Dresden, Dresden, Germany; 3 Acoustics Research Centre, University of Salford, Manchester, United Kingdom; 4 Environmental Research & Innovation Centre, University of Salford, Manchester, United Kingdom; National University of Medical Sciences, PAKISTAN

## Abstract

The study of the perceived affective qualities (PAQs) in soundscape assessments have increased in recent years, with methods varying from in-situ to laboratory. Through technological advances, virtual reality (VR) has facilitated evaluations of multiple locations in the same experiment. In this paper, VR reproductions of different urban sites were presented in an online and laboratory environment testing three locations in Greater Manchester (‘Park’, ‘Plaza’, and pedestrian ‘Street’) in two population densities (empty and busy) using ISO/TS 12913–2 (2018) soundscape PAQs. The studied areas had audio and video recordings prepared for 360 video and binaural audio VR reproductions. The aims were to observe population density effects within locations (Wilcoxon test) and variations between locations (Mann-Whitney U test) within methods. Population density and comparisons among locations demonstrated a significant effect on most PAQs. Results also suggested that big cities can present homogenous sounds, composing a ‘blended’ urban soundscape, independently of functionality. These findings can support urban design in a low-cost approach, where urban planners can test different scenarios and interventions.

## 1. Introduction

Since the publication of the ISO/TS 12913–2 [1], the characterisation of the affective attributes regarding the sonic environment has increased significantly over the years [2–7]. These affective attributes, or Perceived Affective Qualities (PAQs), originated from Axelsson et al. [8] research. They helped to detect the sound qualities of the investigated area, resulting in tools for urban sound management, effective urban planning, and noise control [9]. Studies point out that understanding emotional responses to soundscape supports design decisions [10], a better opportunity to achieve users’ satisfaction [11], and quality of life [12].

Regarding the emotional assessment of the acoustic environments, the work of Axelsson et al. [8] has been the reference for soundscape research. Their model was based on Russell’s circumplex affective model for environments [13]. Axelsson et al. [8] synthesised the semantic scales into a two-dimensional space constructed by pleasantness and eventfulness, which later was adopted as the PAQs in method A of the standard ISO/TS 12913–2 [1]. When rotating these two axes at 45 degrees, their diagonals result in additional dimensions, composed of the mixture related to the pleasant and eventful orthogonal axes. Thus, the standard ISO/TS 12913–2 introduces and describes the resulting eight attributes’ pairs: ‘eventful-uneventful’, ‘pleasant-annoying’, ‘vibrant-monotonous’, and ‘calm-chaotic’. However, this model is still under investigation and validation in other languages through the Soundscape Attributes Translation Project [14]. For instance, soundscape investigators lack consensus in identifying the origins and effects of emotional responses to sounds [4, 15, 16]. To assess these scales, researchers use self-reports, where people perceive these sounds through methods ranging from in-situ experiments to laboratory experiments, including virtual reality (VR).

The main methods for subjective data collection in soundscape studies have been soundwalks, interviews, listening tests, and focus groups [17]. The ISO/TS 12.913–2 suggests the first two methods [1]. However, the systematic review from Engel et al. [17] demonstrated that most recent studies use listening tests with the main topic of ‘soundscape quality’, using semantic differential tools to evaluate the stimuli of parks, squares, shopping areas, and traffic sounds, with students and academic staff as participants [17]. The controlled environment of the experiments happens in acoustically treated rooms with calibrated audio reproduction systems [18]. These studies allow the investigation of various aspects influencing auditory codification and perception [19], guaranteeing purity and control of factors [18], and enabling analyses of complex interactions or distinct effects [20]. In the laboratory, there are several listening experiment modalities, including with and without visual material [21], from simple (mono) [22] to complex audio reproduction (spatial audio) [23], multimodality (different sensorial stimuli), potentially implemented through Virtual Reality (VR) experiments.

Furthermore, VR technology can facilitate the evaluation of multiple locations in the same experiment under safe conditions [18] in a more engaging experiment [24], allowing observations of the effects on presence, realism, involvement, distraction level, and auditory aspect [25]. Participants are immersed in realist scenarios, giving them a ‘sense of presence’ [26], representing a similar experience of being in the real place. Audio, visual, tactile, and smells can enhance the multimodal experience. Regarding the virtual sonic environment, reproduction formats vary from mono to spatial audio [27]. Binaural audio played by headphones and ambisonics audio through loudspeakers are the main forms of audio reproduction in soundscape studies. In Sun et al. [28, 29] study, when testing spatial audio through headphones and loudspeakers in a VR experiment, participants subjective responses demonstrated that the sense of immersion and realism were not affected by the type of audio reproduction.

Nevertheless, field and VR laboratory tests should sustain the experimental ‘ecological validity’. To guarantee this experimental condition, the laboratory reproduction of real-life audiovisual stimuli should create a similar sense of immersion and realism as in the original scenery [30]. If similarities are maintained between real and VR reproductions, laboratory experiments can support research with controlled factors. However, this may amplify results and biased conclusions, thus, outcomes should be interpreted cautiously [6]. So far, most studies have confirmed similar soundscape perceptions between in-situ and laboratory VR listening experiments [6, 31–33], pointing out VR methods as a good strategy for soundscape research.

Another self-report data collection method is online experiments, which increased significantly during COVID-19. For example, the Lucid platform for online data collection in research tripled in purchases from 2019 to 2020 [34]. The drawbacks of online experiments are reduced attentiveness [34], the lack of controlled audio reproductions and system calibration used by the participants [32], the absence of assistants during the experiment, and unreliable responses given by different participants due to their context, among others [35]. The advantages of using a web-based approach in soundscape studies include a higher number of participants, ease of sharing, and engagement of citizens in sound design and urban planning. Regarding the urban sound design, ‘local experts’, people who live and use the studied location [36], local authorities, planners, designers and whoever is related to the site, should discuss their interests to indicate activities to the urban place [37]. Diversity in activities tends to create a more dynamic atmosphere in urban places. In these circumstances, acoustic zoning consists in giving the distance in space, time, or both [37]. Bento Coelho describes in his soundscape design process that a sound catalogue or sound identity map should be developed, where sounds are correlated to functions, activities, other senses, and preferred sounds of the place [38]. Additionally, appropriateness [7], and the expectations [39] of the sonic environment should reach towards a coherent soundscape. The guidelines mentioned above can delimit the acoustic zones based on sound sources, avoiding ‘lo-fi’ soundscapes. The latter represents sounds that are not easily located in an obscure population of sounds [40]—which may represent a ‘blended’ sonic environment. Its opposite is the ‘hi-fi’ soundscape with a clear distinction between foreground and background sounds [40], making it simple to identify the predominant sound source in the sonic environment.

The acoustically delimitated zones can correlate to the characteristics and functions of the locations. Urban soundscape studies have sites varying among natural places, public areas, squares, pedestrian streets, and shopping areas [17]. However, vibrant places are less studied. These are related to pleasant and eventful attributes linked to busy contexts in specific human activities [41]. Previous works confirm that the ‘presence of people’ in places leads to the ‘eventful’ dimension and may define a vibrant experience [3, 29]. Most soundscape studies investigate parks, where natural sounds indicate psychological restoration [42], places for human de-stress [5, 42], and improvement in the sonic environment evaluation [43]. These locations may represent pleasant places that can flourish feelings of joy and facilitate the public into fulfilling self-selected activities.

Based on the presented factors, this work adopts VR experiments through an online VR experiment, The Manchester Soundscape Experiment Online (MCR online), carried out in 2020, and a laboratory VR experiment, The Laboratory VR Soundscape Experiment (LAB VR), carried out in 2022, using spatial audio and 360° video recordings. Participants will be exposed to three urban sites (Peel Park—an urban park; Market Street—a pedestrian street; and Piccadilly Gardens—a plaza) in two population densities (empty and busy), followed by a self-report of the soundscape PAQs. The investigated hypotheses are four statements stated below. The Wilcoxon signed-rank test will be applied for comparisons within the two experiments, empty and busy conditions for the same location. In this case, the null and alternative hypotheses are:

- H_01_ = The perceptual response (PAQs) will change when in different population densities in the same location and experiment; and
- H_a1_ = The perceptual response (PAQs) will not change when in different population densities in the same location and experiment.

The Mann–Whitney U test will be applied to compare the different soundscape locations for each data collection method, being their hypotheses as follows:

- H_02_ = The perceptual response (PAQs) will change according to the different urban locations for each data collection method; and
- H_a2_ = The perceptual response (PAQs) will not change according to the different urban locations for each data collection method.

The PAQs of the ISO/TS 12913–2 [1] were selected as subjective responses given its international standardization. The aim is to observe the PAQ results from the previous two perspectives. The first view concerns an evaluation within each experiment where differences between the two population densities are analysed. Second, the variation between locations for each experimental method is investigated. Findings are considered to enhance comprehension of how people perceive the studied urban soundscape conditions through different VR methods, supporting urban sound design and future urban development appraisal [44].

## 2. Materials and methods

**Fig 1:**
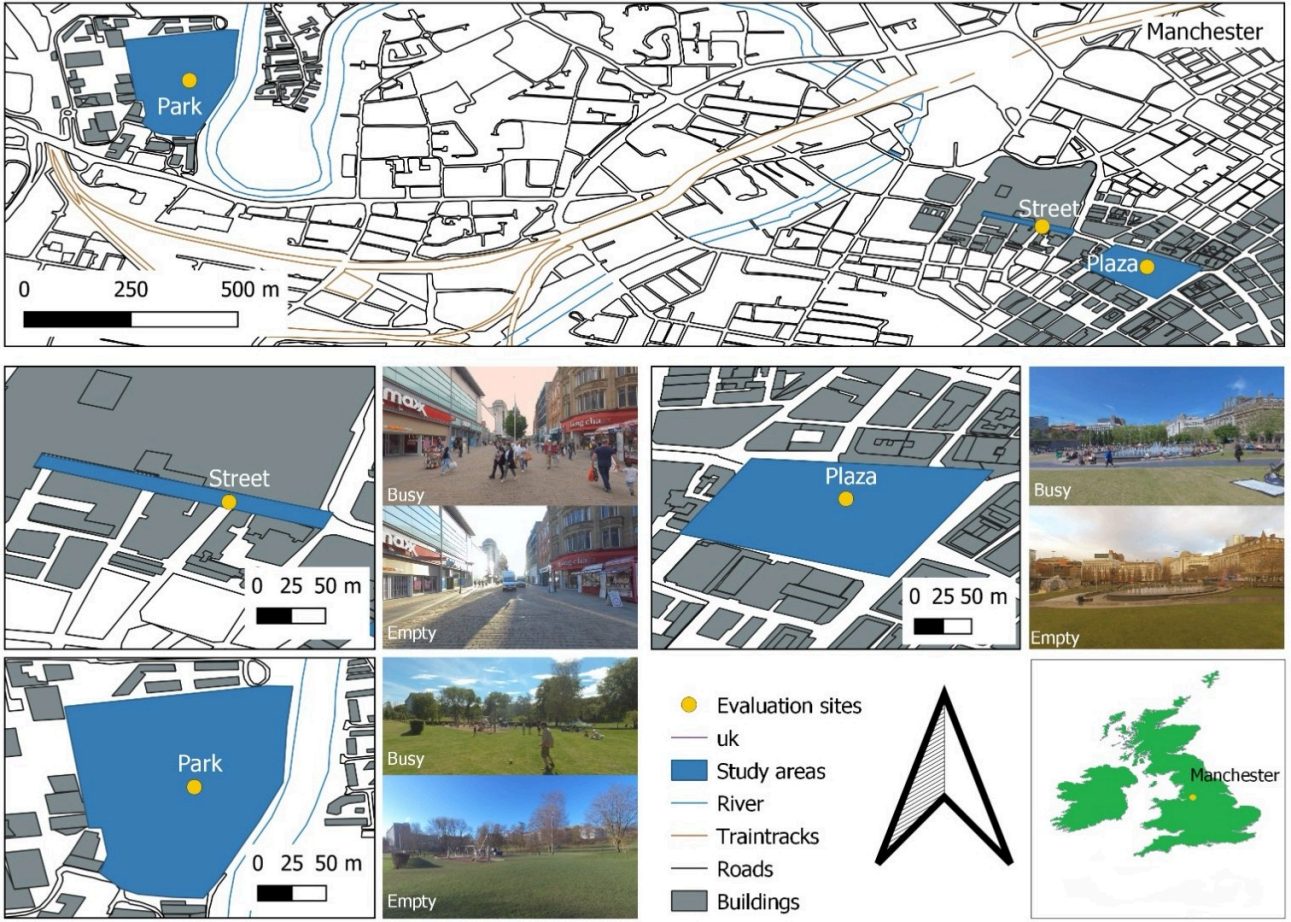
Study areas. The top illustrates all locations on the Manchester map. The middle row shows the ‘Street’ map, pictures of empty and busy conditions, the ‘Plaza’ map, and pictures of empty and busy conditions. The bottom row illustrates the ‘Park’ map, pictures of empty and busy conditions, north, and the UK map with Manchester’s position. The yellow dots are the evaluated sites. The areas shaded in blue are the areas studied. Pictures of Carvalho taken between 2019 to 2020.

Fig 1 illustrates the investigated areas defined according to a previous study by Carvalho et al. [45]. They were derived from a structured interview to identify locations within the four quadrants of the ISO/TS 12913–2 [1] PAQs quadrants (‘vibrant’, ‘calm’, ‘monotonous’, and ‘chaotic’ attributes).

### 2.1 Study areas

Piccadilly Gardens (a popular plaza in the city centre) represented the ‘vibrant’ attribute called ‘Plaza’ from now on in the paper. Peel Park (a park at the University of Salford) exemplified the ‘calm’ attribute referred to as ‘Park’ hereafter. A bus stop (common bus stop in front of the University of Salford) corresponded to the ‘monotonous’ attribute, and Market Street (pedestrian commercial street) was selected for the ‘chaotic’ attribute, hereinafter, referred to as ‘Street’. The bus stop was excluded because the LAB VR experiment did not use this condition.

Piccadilly Gardens is the largest public space in central Manchester, with 1.49 Ha and various functions such as crossing, eating places, children’s play, and places for small and large events [46]. A contemporary design changed the garden into a Plaza in 2002 [46] that included a water fountain, playground, café store, a barrier by Japanese architect Tadao Ando that also served as protection of the central plaza, grass areas, and trees where people sit on sunny days. The location is surrounded by Piccadilly Street at the north, Mosley Street at the west, Parker Street at the south, and One Piccadilly Gardens building at the east side. The constant sound source in both population densities was sounds originating from the water fountain. In the empty condition, the fountain sound was predominant, but mechanical sounds were also present in the background. In the busy condition, the predominant sound was a rich presence of human sounds, such as chat and kids shouting, while traffic sounds from nearby trams and their breaks were audible in the background.

Peel Park has 9.40 Ha and is one of the oldest public parks in the world, dating from 1846 [47]. Today, it integrates with the Peel Park Campus of the University of Salford, including walking paths, tall and scattered trees, a playground structure, sculptures, a garden with flowerbeds, lots of green area, and benches to sit. The park is surrounded by the Student Accommodation and access to the David Lewis Sports Ground at the north; the River Irwell with a bridge to The Meadow, a public green space, and a housing area at the east; the Maxwell Building, and the Salford Museum and Art Gallery on the south; and the University House, the Clifford Library, and the Cockcroft Building at the westside. The local population uses the location for ‘passive’ recreation, exercise, and crossing paths to other sites. The constant sound source in both population densities was sounds of nature, specifically from the calls of birds. In the empty condition, four different bird calls were predominant and identified, them being ‘Pica Pica’, ‘Eurasian Wren’, ‘Redwing’, and the ‘Eurasian Tree Cree’. In the busy conditions, the bird call was not recognized, given the masking effects of human sounds, placing the nature sounds in the background, while the predominant foreground sounds were children talking, shouting, and playing football.

Market Street is approximately 370 meters long, with a 280-meter pedestrian zone occupying around 0.91 Ha. Exchange Street delimits it on the west until High Street on the east. The pedestrian zone is between High Street and Corporation Street, with primarily commercial activities such as clothes and shoe stores, banks, grocery stores, street food huts, gyms, bookstores, mobile stores, pharmacies, coffee stores, and three accesses to the Manchester Arndale Shopping. When the street gains traffic, commercial activities are more related to beauty products, confectionery, stationary, clothing and footwear, coffee shops, and access to the Royal Exchange Building. The constant sound source in both population densities was the ‘hoot’ from the nearby tram. In the empty condition, the predominant sounds were mechanical sounds, such as snaps of machinery in different rhythms and frequency intervals. Traffic and chats were also present in this condition. In the busy condition, snaps were still present, but predominance was related to human-made sounds, such as babble and footsteps.

### 2.2 Audiovisual preparation

**Fig 2:**
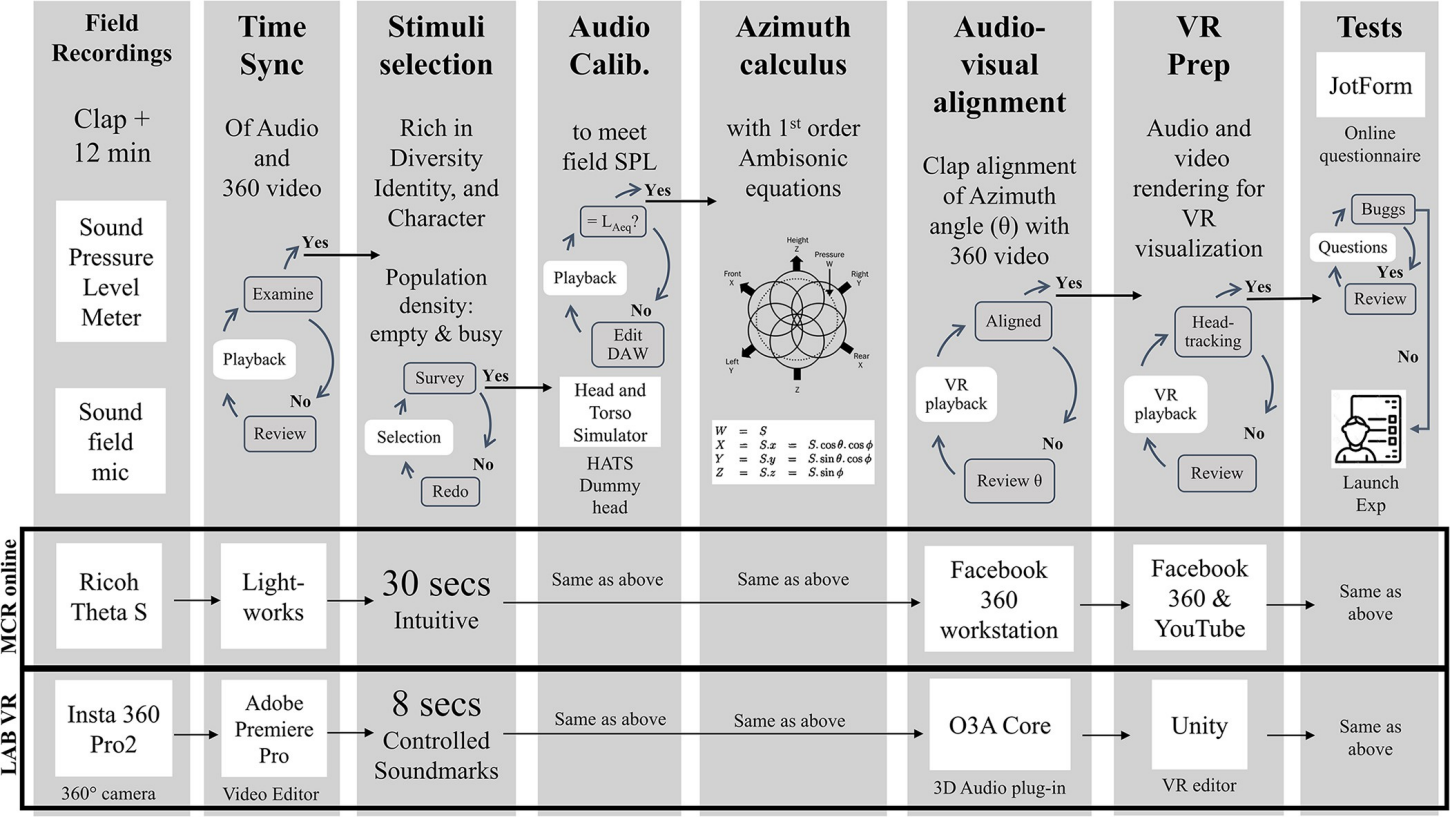
Workflow of audiovisual preparation for launching experiments. Each column represents a stage.

Two different footages of the same studied areas were tested with two methods: an online VR questionnaire (MCR online) and a laboratory VR experiment (LAB VR). Audiovisual stimuli were different recordings in each experiment because participants of the MCR online complained about the video resolution. Thus, new recordings with a higher resolution camera occurred for the LAB VR. Nevertheless, all recordings were done in the same position. The study was conducted and approved by the Research, Innovation and Academic Engagement Ethical Approval Panel of the University of Salford (protocol code STR1819-31). Fig 2 illustrates the workflow for constructing the VR environments for the experiments.

The Sound field microphone ST250 and the sound pressure level meter, type BSWA 308, were used in recordings with a sampling rate of 44.1 kHz. For the MCR online, the microphone was plugged into a ZOOM H6 Handy Recorder for the audios, and the Ricoh Theta S camera was used for the 360° videos. In the LAB VR, the microphone was plugged into an Edirol R44 Recorder, and an Insta 360 Pro2 360° video camera was used for video recording.

Given ethical approval restrictions, a sign warning ‘Filming in progress’ was displayed with the equipment for public awareness before recordings. With a previously calibrated sound pressure level meter, a one-minute sample of A-weighted equivalent continuous sound pressure (L_Aeq,60_) registered sound levels to adjust the field levels to laboratory reproductions. After initiating the microphone and camera, the researcher clapped in front of the equipment for future audiovisual alignment.

Recordings were done in the early hours (4 to 6 am) of a weekday for empty, and the afternoon (2 to 4 pm) at the weekend for busy conditions. On arrival, the locations were established so, as to not interrupt circulation. The experimenter merged into the scenery, and the recordings lasted 10 to 12 minutes [29]. These procedures resembled those done by the ‘Urban Soundscapes of the World’ project group [28, 29, 48].

Video files were transformed into equirectangular format (MCR online) or edited together (VR LAB). Audio and video stimuli were synchronised in time with the initial clap, verified and corrected when necessary. On the MCR online, the selected audiovisual stimuli had a 30-second duration following a previous study [49]. The stimuli duration changed to 8 seconds in the LAB VR, using as reference a fMRI soundscape experiment [50], because of a physiological test in another stage of the experiment.

A population density calculation occurred using the footage to select the audiovisual stimuli. The people-counting criteria followed a previous study that measured the number of individuals from a selected frame [51]. Surveys with ten participants were used to certify selected footage for empty and busy conditions. When the criteria failed, new stimuli selection took place. A descriptive analysis of the sound events, foreground and background sounds, was done of the footage with empty and busy conditions to select fragments rich in soundscape diversity [52], identity [53], character [54], and sound signal [40]. The LAB VR also had controlled sound signals, such as the water fountain at the ‘Plaza’, the tram hoot at the ‘Street’, and the bird calls at the ‘Park’ in empty and busy conditions.

Audio files were calibrated to the field sound levels using a pre-calibrated High-frequency Head and Torso Simulator (HATS) connected to a PULSE software of Brüel & Kjær [6]. Audiovisual stimuli were aligned through audio rotation using the azimuth angle θ from the first-order ambisonics equations, that is, audio X from front-back positions of B-format audio recordings—WXYZ) [22]. The audio and video files were rendered into 3D head-tracked stimuli for VR reproduction. Stimuli reproductions were tested through the final experimental VR and headphone setup, recorded for calibration, verified in each step, and corrected when necessary.

### 2.3 Participants and experimental procedures

Participants were recruited by the Acoustics Research Centre of Salford mailing list representing people with connections to the University of Salford, and above 18 years old in both experiments. The MCR online also had respondents recruited by convenience sampling over the internet on social networks, such as Facebook, Instagram, Twitter, and LinkedIn, and participated voluntarily from August 26 to November 30, 2020. The LAB VR received a compensation of £25 in Amazon voucher. These subjects were recruited from June 27 to August 5, 2022.

Conditions were three locations (‘Park’, ‘Plaza’, and ‘Street’) in two population densities (empty and busy) responding to the eight PAQs questions. MCR online had 80 individuals rating the ‘Plaza’ and ‘Street’ (80 x 2-sites x 2-densities x 8-PAQs = 2560 results), and 75 assessing the ‘Park’ (75 x 2-densities x 8-PAQs = 1200 results). LAB VR had 36 participants (36 x 3-sites x 2-densities x 8-PAQs = 1728 results).

At the beginning of both experiments, participants signed a written consent form and received an information sheet describing the experiment and its procedure. Given the MCR online also had Brazilian participants, the questionnaires were translated to the Portuguese language. Subjects were divided into two groups to reduce experimental time: ‘Plaza’ and ‘Street’, and ‘Park’ and a bus stop. Recommendations were to use headphones and, when using mobile phones, to turn into a landscape orientation for better performance.

In the LAB VR, tests were done inside a semi-anechoic chamber at the Acoustics Research Centre of the University of Salford, Manchester, UK. Considering that cases of COVID were still occurring (July 2022), an email detailed COVID-free protocol before arriving. Participants sat in the centre of the semi-anechoic chamber, watched a short video explaining the research, answered the general information questions, and conducted a training session. They watched the six audiovisual stimuli through the VIVE HMD with a Beyerdynamic DT 1990 Pro headset as many times as they wished and answered the subjective questions presented on a laptop.

Questionnaires were developed in an online platform. For the MCR online, the questionnaire began with a written consent form. General questions were asked about demographics (gender, age, nationality, and residency), auditory health (evidence of hearing loss, and tinnitus), and digital settings (what audio and video system they used during the experiment). Questions were responded to after watching each video. They were phrased: ‘Please, slide to the word that best describes the sounds you just heard. To the left (-) is NEGATIVE, and to the right (+) is POSITIVE.’ Paired PAQs presented with three synonyms each were ‘unpleasant-pleasant’, ‘uneventful-eventful’, ‘chaotic-calm’, and ‘monotonous-vibrant’ PAQs. Scores ranged from -10 to +10 for negative to positive semantic values of terms through a slider.

In the LAB VR, video and questions were randomly presented. General questions were demographic, auditory health (as in the MCR online), number of languages spoken, education level, and acoustic or music background (no, a little, moderate, and expert level). The experimental questions were formulated: ‘To what extent do you think the sound environment you just experienced was. . . 0 = Not at all, 50 = Neutral, and 100 = Extremely’. The PAQs were presented individually and rated through a slider. The soundscape attributes tested were ‘pleasant’, ‘calm’, ‘uneventful’, ‘monotonous’, ‘annoying’, ‘chaotic’, ‘eventful’, and ‘vibrant’ PAQs separately. In both experiments, there was a final open question to have feedback regarding experiments.

### 2.4 Statistical analysis

Since data collection had different scales, the MCR online results separated the Paired PAQs, and -10 to +10 ratings inverted to zero (0) to one hundred (100) scores, while the LAB VR maintained as in the original scale. A summary of collected data is presented in Table 1. Statistical analysis included the Wilcoxon signed-rank test for comparisons of the empty and busy conditions within the same location, and the Mann–Whitney U test for comparing the different locations for the same population density, being both tests within the same experiment. Given comparisons were only between two conditions and data collection was on a continuous scale, a correction for multiple comparisons (Bonferroni) was unnecessary. Significant group differences were tested with the help of the statistical package IBM SPSS Statistics 29.0.1.0 ®.

**Table 1:** Summary of the conditions.

Condition	Location	Density	Experiment	n^a^	Month	Year	Season	COVID	
								Rec^b^	Exp^c^
1	Park	empty	MCR online	75	October	2019	Autumn	1	2
2			LAB VR	36	February	2021	Winter	3	3
3		busy	MCR online	75	September	2019	Autumn	1	2
4			LAB VR	36	June	2021	Summer	3	3
5	Plaza	empty	MCR online	80	June	2020	Summer	1	2
6			LAB VR	36	December	2020	Winter	2	3
7		busy	MCR online	80	June	2020	Summer	1	2
8			LAB VR	36	June	2021	Summer	2	3
9	Street	empty	MCR online	80	October	2019	Autumn	1	2
10			LAB VR	36	February	2021	Winter	2	3
11		busy	MCR online	80	June	2019	Summer	1	2
12			LAB VR	36	July	2021	Summer	2	3

^a^Number of participants (n)

^b^Recordings (Rec), and

^c^Execution of experiments (Exp) before (1), during (2) or with low rates of COVID (3).

## 3. Results

### 3.1 Descriptive analysis of participants

Table 2 presents the demographic information for the MCR online and LAB VR experiments. The MCR online occurred online from August to November 2020. The 155 participants came from 63 countries: 52% from Brazil, 12% from the UK, and 14% from other parts of the world, including Europe, Africa, North and South America, Asia, and the Middle East. In Group 1, 80% used a computer screen and 20% a smartphone to watch the videos, while 76% used headphones and 24% external audio to reproduce audio signals during the experiment. 89% declared they had no hearing loss, and 11% had some hearing loss. 77% mentioned not to have tinnitus, and 23% to have signs of tinnitus [45]. In Group 2, 86% used a computer screen and 14% a smartphone to watch the videos, while 65% used headphones and 35% external audio to reproduce audio signals during the experiment. 90% declared they had no hearing loss, and 10% had some hearing loss. 81% mentioned not to have tinnitus, and 19% to have signs of tinnitus [55].

**Table 2:** Summary of the demographic information of the MCR online and LAB VR experiments.

Experiment	n^a^	Gender			Age in years			
		Women	Males	Prefer not to say, or non-binary	Range	Mean	SD^b^	Majority
MCR online								
Group 1	75	49%	48%	3%	21–68	37	12	26–35 (40%)
Group 2	80	51%	45%	4%	21–67	38	11	26–35 (35%)
LAB VR	36	36%	64%	0%	19–60	32	10	26–35 (33%)

^a^Number of participants (n), and

^b^Standard deviation (SD).

For the LAB VR, participants originated from 11 countries, with 47% from the United Kingdom, 17% from India, and 36% from other parts of the world including Europe, Africa, South America, and Asia. 97% declared no hearing loss, and 3% mild hearing loss. 83% mentioned not having tinnitus, and 17% heard infrequently or regularly signs of tinnitus.

The MCR online counted 4.3 times more participants (N = 155) compared to the LAB VR (N = 36). In summary, over 50% of Brazilians participated in the MCR online, followed by 12% of British with a predominant age range of 26 to 35 years old (35%) and balanced gender distribution.

### 3.2 Descriptive analysis of auditory stimuli

The acoustic and psychoacoustic characteristics of the auditory stimuli for each tested scenario are demonstrated in Tables 3 and 4. For the MCR online, 17 visits from January to December 2019 on days with no precipitation were done at Peel Park, Piccadilly Gardens, and Market Street in empty and busy conditions to collect audio recordings for the online experiment. For the LAB VR, a total of nine visits to execute field recordings were done from December 2020 to July 2021 on days with no precipitation forecast in the empty and busy conditions at Piccadilly Gardens (Plaza), Market Street (Street), and Peel Park (Park).

**Table 3:** Field one-minute sample of A-weighted equivalent continuous sound pressure (L_Aeq,1min_) in dB(A).

Scenario	MCR online L_Aeq,1min_ dB(A)	LAB VR L_Aeq,1min_ dB(A)
Park empty	46	47.1
Park busy	56	53.9
Plaza empty	50	64.5
Plaza busy	70	64.3
Street empty	60	55.1
Street busy	69	62.8

**Table 4:** Psychoacoustic metrics for the LAB VR audio stimuli. Loudness (N), Sharpness (S), Roughness (R), Fluctuation Strength (FS), and Tonality (T).

Parameter	‘Plaza’ empty	‘Plaza’ busy	‘Park’ empty	‘Park’ busy	‘Street’ empty	‘Street’ busy
N [sone]	16.82	23.01	13.46	15.09	10.61	18.13
S [acum]	1.70	1.84	1.33	1.31	1.31	1.43
R [asper]	0.02	0.02	0.03	0.03	0.02	0.03
FS [vacil]	0.02	0.03	0.03	0.03	0.02	0.04
T Avg Arit [tu]	0.06	0.25	0.13	0.25	0.02	0.11

As observed in Table 3, the higher value for 1 min L_Aeq_ on the MCR online was for the ‘Plaza’ busy scenario with 70 dB(A), while the smallest value was observed for the ‘Park’ empty scenario with 46 dB(A). In the LAB VR, the superior value was for the ‘Plaza’ empty with 64.5 dB(A), and the smallest appeared for the ‘Park’ empty scenario with 47.1 dB(A).

Table 4 shows the psychoacoustic metrics of each scenario’s auditory stimuli used for the LAB VR. Greater values are observed at the ‘Plaza’ busy for Loudness (N = 23.01 sone), Sharpness (S = 1.84 acum), and Tonality (T = 0.25 tu); at the ‘Park’ empty for Roughness (R = 0.03 asper); at the ‘Park’ busy for Roughness (R = 0.03 asper) and Tonality (T = 0.25 tu); and at the ‘Street ‘ busy for Roughness (R = 0.03 asper) and Fluctuation Strength (FS = 0.04 vacil). The smallest values are observed at the ‘Street’ empty for Loudnes (N = 10.61 sone), Sharpness (S = 1.31 acum), Roughness (R = 0.02 asper), Fluctuation Strength (FS = 0.02 vacil), and Tonality (T = 0.02 tu). It was also observed the smaller values of Sharpness(S = 1.31 acum) at the ‘Park’ busy, Roughness (R = 0.02 asper), at the ‘Plaza’ busy; Roughness (R = 0.02 asper), and Fluctuation Strength (FS = 0.02 vacil) at the ‘Plaza’ empty.

### 3.3 Wilcoxon signed-ranks test results for busy versus empty conditions

**Fig 3:**
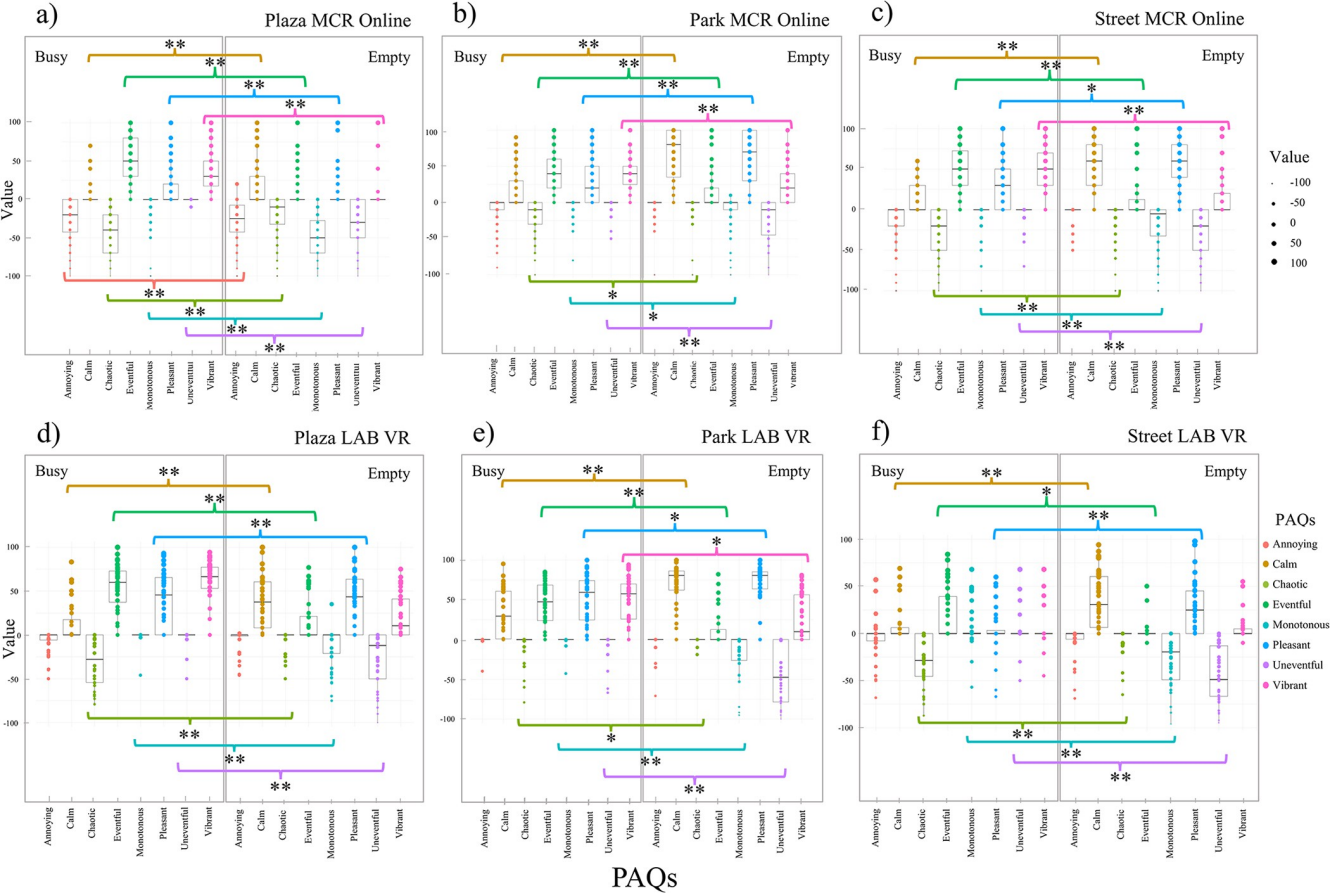
Boxplots comparing empty and busy conditions within the same experiment using the Wilcoxon signed-rank test. Columns for ‘Plaza’ (3a & 3d), ‘Park’ (3b & 3e), and ‘Street’ (3c & 3f); and rows for MCR online (3a-3c), and LAB VR (3d-3f). * for significant p-value at < .05, and ** for significant p-value at < .001.

The Wilcoxon signed-ranks test evaluated how the spaces were rated in busy and empty conditions for each location and the data collection method. Table 5 shows the Wilcoxon signed-ranks test results, which suit two related samples with a non-normal distribution. Values with significant p-values indicate that there are differences between samples. 85.4% (41 PAQs) of results presented significant differences between empty and busy conditions in the studied locations, and 14.6% (7 PAQs) of results had an unexpected similarity. Fig 3 shows a set of boxplots for each studied area and data collection method, where comparing the results in busy and empty conditions is possible. It also represents the significance level of the Wilcoxon signed rank test using * for p-values below 0.05 and ** for p-values inferior to 0.001. In the boxplots, there is a higher distribution in busy conditions on positive qualities such as ‘calm’, ‘eventful’, ‘pleasant’ and ‘vibrant’ in all samples (3a-3f), while in empty conditions, ratings concentrated over the neutral answer. A smaller distribution of negative qualities such as ‘uneventful’ and ‘monotonous’ is also observed.

**Table 5:** Wilcoxon signed-rank test results for the comparison of busy vs. empty conditions. Where * represents the p-value for 2-tailed significance.

Method	MCR Online						LAB VR					
Location	Street		Park		Plaza		Street		Park		Plaza	
**PAQs** **busy vs.** **empty**	Z	p-value*	Z	p-value*	Z	p-value*	Z	p-value*	Z	p-value*	Z	p-value*
Chaotic	-4.626	0.000	-3.082	0.002	-3.967	0.000	-3.99	0.000	-2.397	0.017	-3.608	0.000
Calm	-3.503	0.000	-6.645	0.000	-6.531	0.000	-3.817	0.000	-4.227	0.000	-3.909	0.000
Annoying	-0.958	0.338	-1.892	0.058	-3.685	0.000	-0.448	0.654	-0.339	0.735	-0.259	0.796
Pleasant	-2.227	0.026	-5.791	0.000	-5.02	0.000	-3.359	0.001	-2.867	0.004	-0.049	0.961
Uneventful	-6.647	0.000	-4.93	0.000	-5.756	0.000	-4.577	0.000	-4.192	0.000	-4.012	0.000
Eventful	-7.160	0.000	-4.965	0.000	-6.903	0.000	-3.02	0.003	-4.263	0.000	-4.535	0.000
Monotonous	-6.629	0.000	-2.262	0.024	-4.557	0.000	-4.229	0.000	-3.414	0.001	-2.442	0.015
Vibrant	-6.375	0.000	-3.713	0.000	-6.888	0.000	-1.252	0.210	-3.029	0.002	-4.611	0.000

As observed in Table 5, the significant results for the MCR Online dataset between busy and empty presented in descending order were as follows: the ‘eventful’ PAQ in the ‘Street’ (Z = -7.16, p<0.001); the ‘vibrant’ PAQ in the ‘Plaza’ (Z = -6.888, p<0.001); the ‘uneventful’ PAQ in the ‘Street’ (Z = -6.647, p<0.001); the ‘calm’ in the ‘Park’ (Z = -6.645, p<0.001); the ‘monotonous’ PAQ in the ‘Street’ (Z = -6.629, p<0.001); the ‘pleasant’ PAQ in the ‘Park’ (Z = -5.791, p<0.001); the ‘chaotic’ PAQ in the ‘Street’ (Z = -4.626, p<0.001); and the ‘annoying’ PAQ in the ‘Plaza’ (Z = -3.685, p<0.001).

As observed in Table 5, the PAQ with non-significant values on the MCR Online and LAB VR is the quality of ‘annoying’ with a score of zero in all studied areas, except on the MCR Online at the ‘Plaza’. Non-significant level ratings regarding the quality ‘pleasant’ were observed with a score around 50 at the ‘Plaza’ and ‘vibrant’ with a neutral score at the ‘Street’ studied areas. The non-significant p-values from the qualities mentioned above indicate no perceived acoustic differences between the empty and busy conditions.

For the LAB VR dataset, the superior difference between busy and empty were in descending order as follows: the ‘vibrant’ PAQ at the ‘Plaza’ (Z = -4.611, p<0.001); the ‘uneventful’ PAQ at the ‘Street’ (Z = -4.577, p<0.001); the ‘eventful’ PAQ at the ‘Park’ (Z = -4.263, p<0.001); the ‘monotonous’ PAQ at the ‘Street’ (Z = -4.229, p<0.001); the ‘calm’ PAQ at the ‘Park’ (Z = -4.227, p<0.001); the ‘chaotic’ PAQ at the ‘Street’ (Z = -3.99, p<0.001); and the ‘pleasant’ PAQ at the ‘Street’ (Z = -3.359, p<0.05).

### 3.4 Mann-Whitney U test results for comparison between locations

**Fig 4:**
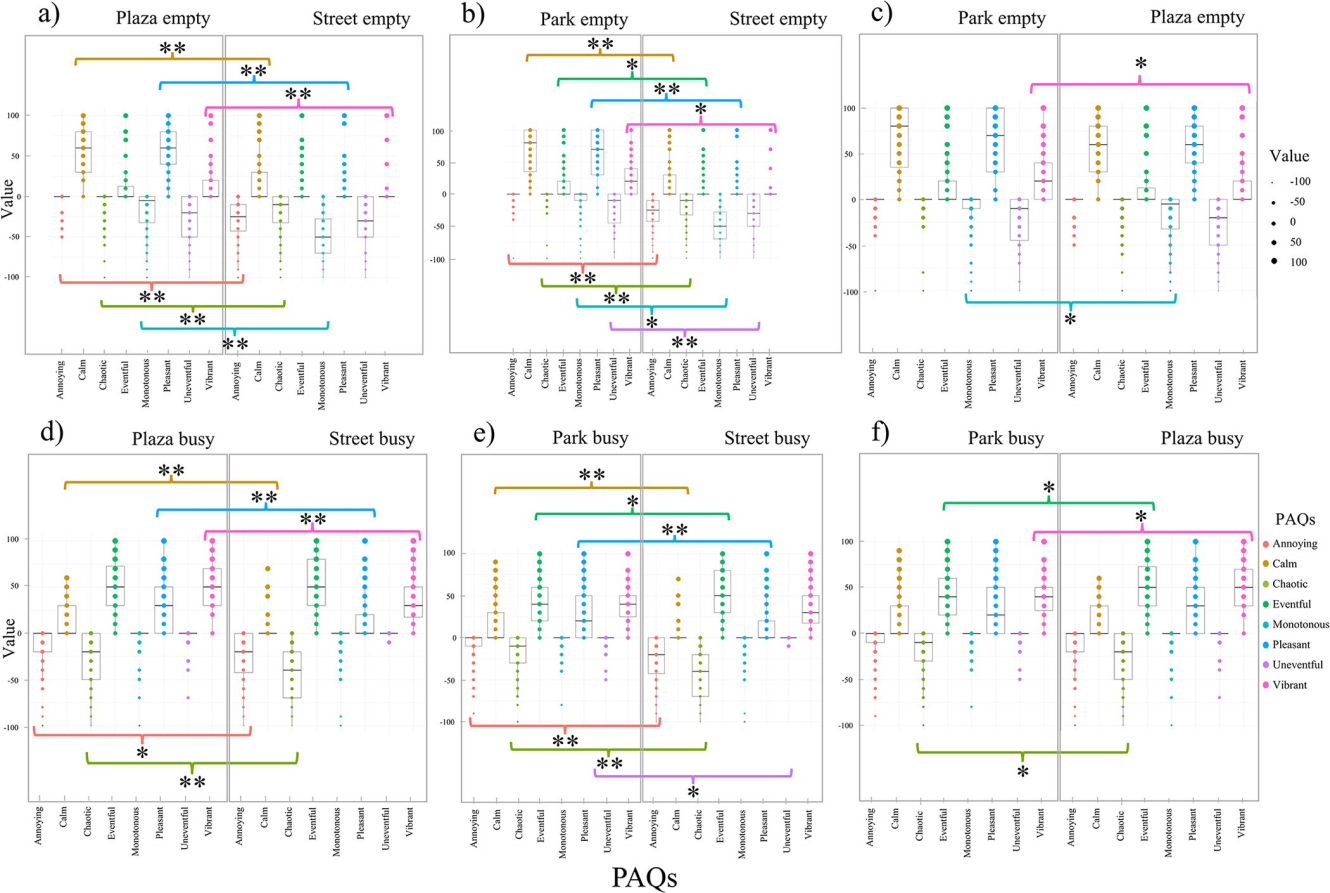
Boxplots comparing different locations for the MCR online, using the Mann-Whitney U test. Columns for comparisons of ‘Plaza’ vs. ‘Street’ (4a & 4d), Park’ vs. ‘Street’ (4b & 4e), and ‘Park’ vs. ‘Plaza’ (4c & 4f); and rows for empty (4a-4c), and busy (4d-4f) conditions. * for significant p-value at < .05, and ** for significant p-value at < .001.

**Fig 5:**
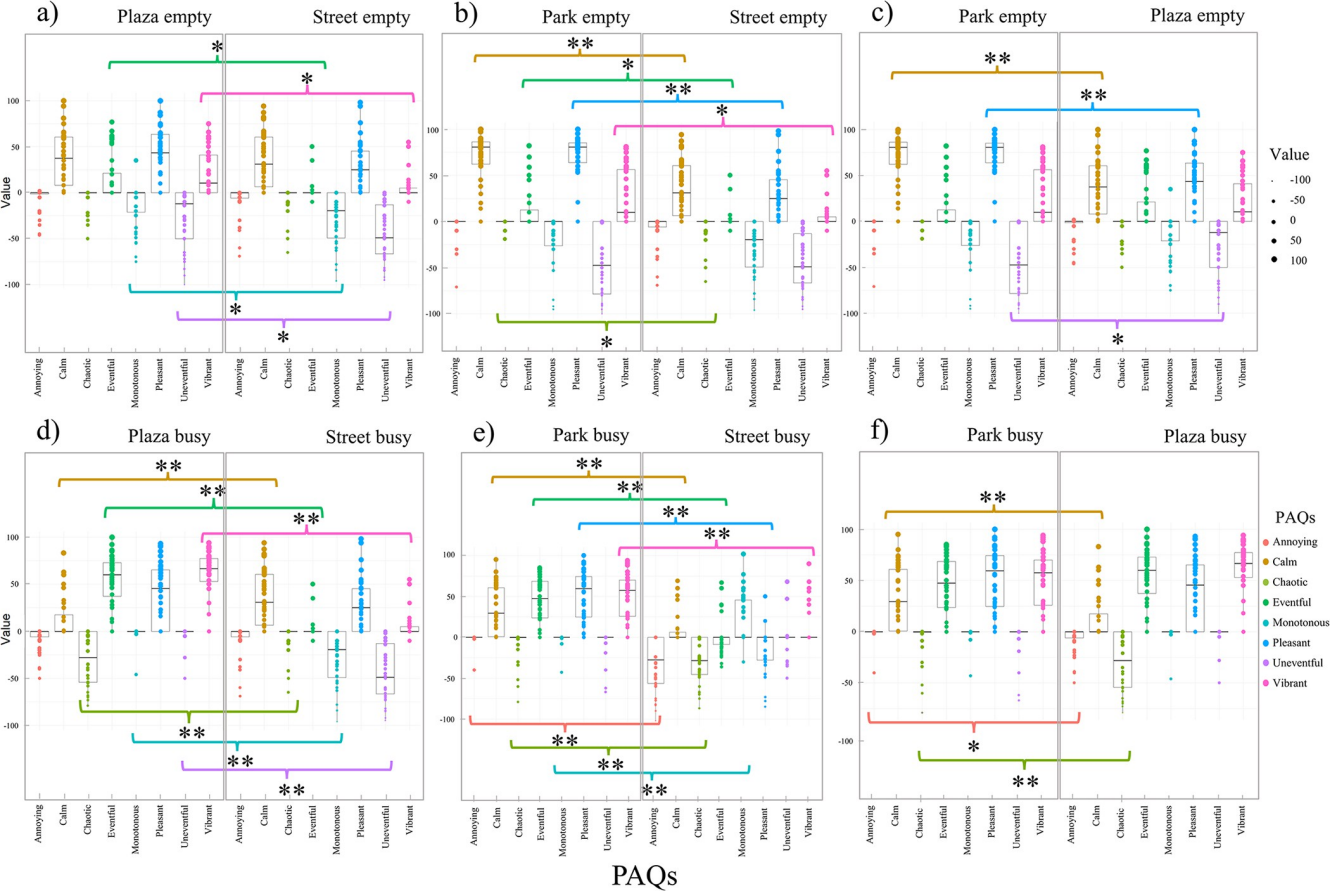
Boxplots comparing different locations for the LAB VR, using the Mann-Whitney U test. Columns for comparisons of ‘Plaza’ vs. ‘Street’ (5a & 5d), Park’ vs. ‘Street’ (5b & 5e), and ‘Park’ vs. ‘Plaza’ (5c & 5f); and rows for empty (5a-5c), and busy (5d-5f) conditions. * for significant p-value at < .05, and ** for significant p-value at < .001.

The Mann-Whitney U test helped compare the same population density condition among different locations in each data collection method. Table 6 shows the results of the Mann-Whitney U test, which suits two independent samples with non-normal distribution. Significant p-values indicate that there are differences between locations. Some PAQs had no differences among locations, meaning no significance with a p-value higher than 0.05. Figs 4 and 5 show the set of boxplots for each studied area comparisons and data collection, where it is possible to compare the results in busy and empty conditions. It also represents the significance level of the Mann-Whitney U tests using * for p-values below 0.05 and ** for p-values inferior to 0.001.

**Table 6:** Results of the Mann-Whitney U test for comparisons between sites in each experiment. Where * represents the p-value for 2-tailed significance.

MCR Online												
Conditions	‘Street’ vs. ‘Park’ busy 19 x 10		‘Street’ vs. ‘Park’ empty 17 x 8		‘Plaza’ vs. ‘Street’ busy 16 x 19		‘Plaza’ vs. ‘Street’ empty 14 x 17		‘Plaza’ vs. ‘Park’ busy 16 x 10		‘Plaza’ vs. ‘Park’ empty 14 x 8	
Perceived Acoustic Quality (PAQ)	U	p-value*	U	p-value*	U	p-value*	U	p-value*	U	p-value*	U	p-value*
chaotic	1432.5	0.000	1893.5	0.000	2254.5	0.001	2123.5	0.000	2471.5	0.049	2916.5	0.615
calm	2163.5	0.000	978.0	0.000	2405.0	0.001	1267.0	0.000	2875.5	0.614	2481.0	0.061
annoying	1852.0	0.000	979.5	0.000	2351.0	0.002	915.0	0.000	2733.0	0.236	2871.5	0.301
pleasant	1946.0	0.000	589.0	0.000	2051.0	0.000	532.0	0.000	2934.0	0.809	2724.5	0.320
uneventful	2754.5	0.022	2356.5	0.018	3038.5	0.094	2901.5	0.299	2905.0	0.463	2707.5	0.277
eventful	2209.0	0.004	2374.0	0.008	3183.5	0.955	2924.5	0.232	2200.0	0.004	2606.0	0.107
monotonous	2898.0	0.555	960.0	0.000	2959.0	0.152	1569.0	0.000	2871.0	0.394	2424.0	0.022
vibrant	2637.0	0.190	1390.0	0.000	2148.5	0.000	2120.0	0.000	2373.5	0.024	2230.5	0.003
**LAB VR Experiment**												
**Conditions**	**‘Street’ vs. ‘Park’** **busy 25 x 23**		**‘Street’ vs. ‘Park’** **empty 24 x 22**		**‘Plaza’ vs. ‘Street’busy 21 x 25**		**‘Plaza’ vs. ‘Street’** **empty 20 x 24**		**‘Plaza’ vs. ‘Park’** **busy 21 x 23**		**‘Plaza’ vs. ‘Park’** **empty 20 x 22**	
**Perceived Acoustic** **Quality (PAQ)**	U	p-value*	U	p-value*	U	p-value*	U	p-value*	U	p-value*	U	p-value*
chaotic	331.0	0.000	536.5	0.037	326.0	0.000	635.0	0.837	363.5	e	553.0	0.063
calm	305.0	0.000	260.5	0.000	333.5	0.000	644.0	0.964	357.5	0.001	259.0	0.000
annoying	339.5	0.000	528.0	0.057	641.5	0.928	595.5	0.461	506.5	0.021	580.0	0.268
pleasant	52.5	0.000	211.0	0.000	513.0	0.123	512.5	0.122	504.5	0.105	269.5	0.000
uneventful	616.5	0.608	621.0	0.759	137.0	0.000	452.0	0.025	609.5	0.427	473.5	0.044
eventful	129.0	0.000	517.0	0.038	91.0	0.000	442.5	0.003	522.0	0.155	580.0	0.361
monotonous	365.0	0.000	502.0	0.080	278.0	0.000	457.5	0.021	631.0	0.664	589.5	0.453
vibrant	193.0	0.000	419.0	0.005	88.0	0.000	408.5	0.004	480.5	0.059	620.0	0.743

For MCR online, 64.6% (31 PAQs) of results presented significant differences when comparing different locations, and 35.4% (17 PAQs) had similar results. Fig 4 shows the results from MCR online. It is possible to observe in the comparison of ‘Plaza’ vs. ‘Street’ that in the empty condition, there is a higher dispersion of results on the attribute ‘calm’ (Fig 4A). In contrast, in busy conditions, the same dispersion occurs on ‘vibrant’, ‘eventful’, ‘annoying’, ‘chaotic’, and ‘pleasant’ (Fig 4D). For the ‘Park’ vs. ‘Street’ comparison, the dispersion of responses in the empty condition happens on the ‘calm’, ‘monotonous’, and ‘uneventful’ attributes (Fig 4B), meanwhile, for the busy condition dispersion was on the ‘eventful’, ‘pleasant’, ‘vibrant’, ‘annoying’, and ‘chaotic’ attributes (Fig 4E). In the ‘Park’ vs. ‘Plaza’ comparison, the attributes with superior dispersion on the empty condition are ‘calm’, ‘monotonous’, and ‘uneventful’ (Fig 4C), while in the busy condition on the ‘eventful’, ‘vibrant’, ‘annoying’, and ‘chaotic’ attributes (Fig 4F).

Derived from Table 6, the significant U values for each location comparison are presented in descending order. In the MCR Online dataset, the greatest differences between population density were as follows: for ‘Street’ vs. ‘Park’ busy, the ‘uneventful’ PAQ (U = 2754.5, p<0.05); for ‘Plaza’ vs. ‘Park’ busy, the ‘chaotic’ PAQ (U = 2471.5, p<0.05); for the same locations in the empty condition, the ‘monotonous’ PAQ (U = 2424.0, p<0.05); in the ‘Plaza’ vs. ‘Street’ busy, the ‘calm’ PAQ (U = 2405.0, p<0.05); and in the ‘Street’ vs. ‘Park’ empty, the ‘eventful’ PAQ (U = 2374.0, p<0.05).

Regarding the non-significant results also presented in Fig 4 for the MCR online, ratings around zero were observed in different PAQs, as follows: ‘uneventful’ in the ‘Plaza’ vs. ‘Street’ (Fig 4D), and ‘Park’ vs. ‘Plaza’ (Fig 4F) both for the busy condition; ‘eventful’ in the ‘Plaza’ vs. ‘Street’ (Fig 4A), and ‘Park’ vs. ‘Plaza’ (Fig 4C) both for the empty condition; ‘annoying’ for the ‘Park’ vs. ‘Plaza’ (Fig 4C and 4F) in both conditions; ‘calm’ in the ‘Park’ vs. ‘Plaza’ (Fig 4C) for the empty condition; and ‘chaotic’ in the ‘Park’ vs. ‘Plaza’ (Fig 4C) for the empty condition. Additionally, the ‘eventful’ scale had similar scores of around 50 for the ‘Plaza’ vs. ‘Street’ (Fig 4D) in the busy conditions. For the ‘uneventful’ scale, the comparisons of ‘Plaza’ vs. ‘Street’ (Fig 4A), and ‘Park’ vs. ‘Plaza’ (Fig 4C) in the empty condition had values around 20. The ‘pleasant’ PAQ scores were around 60 and 25 in the ‘Park’ vs. ‘Plaza’ for the empty (Fig 4C) and busy (Fig 4F) conditions, respectively. The ‘calm’ scores were around 60 in the ‘Park’ vs. ‘Plaza’ Fig 4C) in the empty condition. For the busy condition, the ‘vibrant’ scores were around 25 in the ‘Park’ vs. ‘Plaza’ (Fig 4F).

For the LAB VR, 62.5% (30 PAQs) of results presented significant differences when comparing different locations, and 37.5% (18 PAQs) had similar results. Fig 5 shows the results from the LAB VR. Regarding the ‘Plaza’ vs. ‘Street’ comparison, the dispersion occurs on the attributes ‘calm’, ‘monotonous’, and ‘uneventful’ for the empty condition (Fig 5A); and ‘pleasant’, ‘eventful’, ‘vibrant’, ‘annoying’, and ‘chaotic’ on the busy condition (Fig 5D). In the ‘Park’ vs. ‘Street’ comparison, the dispersion of results occurs on the attributes ‘calm’, ‘monotonous’, and ‘ uneventful’ in the empty (Fig 5B), while ‘vibrant’, ‘chaotic’, and ‘annoying’ in the busy condition (Fig 5E). Finally, in the ‘Park’ vs. ‘Plaza’ comparison, the attributes with higher dispersion in the empty condition are ‘calm’, ‘pleasant’, ‘monotonous’, and ‘uneventful’ (Fig 5C). In the busy condition (Fig 5F), the dispersion was observed in ‘eventful’, ‘vibrant’, ‘annoying’, and ‘chaotic’ scales.

Derived from Table 6, the significant U values for each location comparison are presented in descending order as follows: the ‘chaotic’ PAQ in the ‘Street’ vs. ‘Park’ empty (U = 563.0, p<0.05); the ‘annoying’ PAQ in the ‘Plaza’ vs. ‘Park’ busy (U = 506.5, p<0.05); the ‘uneventful’ PAQ in the ‘Plaza’ vs. ‘Park’ empty (P = 473.5, p<0.05); the ‘monotonous’ PAQ in the ‘Plaza’ vs. ‘Street’ empty (U = 457.5, p<0.05); the ‘monotonous’ PAQ in the ‘Street’ vs. ‘Park’ busy (U = 365.0, p<0.001); and the ‘calm’ PAQ in the ‘Plaza’ vs. ‘Street’ busy (U = 333.5, p<0.001).

Meanwhile, the non-significant results also noticed in Fig 5, ratings around zero were observed in different PAQs, as follows: ‘uneventful’ in the ‘Park’ vs. ‘Street’ (Fig 5E), and ‘Park’ vs. ‘Plaza’ (Fig 5F) both in the busy condition; ‘monotonous’ in the ‘Park’ vs. ‘Plaza’ for both conditions (Fig 5C and 5F); ‘chaotic’ in the ‘Plaza’ vs. ‘Street’ empty; and ‘eventful’ in the ‘Plaza’ vs. ‘Park’ empty. Four out of six location comparisons had around zero scores for the ‘annoying’ attribute: the ‘Street’ vs. ‘Park’ empty, the ‘Plaza’ vs. ‘Park’ empty, and the ‘Plaza’ vs. ‘Street’ in both conditions (Fig 5A and 5D). Two comparisons scored around 50 for the ‘pleasant’ and ‘eventful’ scales in the ‘Park’ vs. ‘Plaza’ busy (Fig 5F). The two comparisons scored around 40 for the ‘calm’ attribute in the ‘Plaza’ vs. ‘Street’ empty (Fig 5A), and the ‘pleasant’ scale in the ‘Plaza’ vs. ‘Street’ busy (Fig 5D). A score of around 30 appeared for ‘pleasant’ in the ‘Plaza’ vs. ‘Street’ empty (Fig 5A). Meanwhile, the ‘uneventful’ score in the ‘Park’ vs. ‘Street’ for the empty condition (Fig 5B) was around -50, the ‘vibrant’ scale was around 10, and 60 in the ‘Park’ vs. ‘Plaza’ for the empty (Fig 5C), and busy conditions (Fig 5F), respectively.

## 4. Discussion

When verifying the hypothesis (H_01_) regarding different population densities at the same site and experiment, the Wilcoxon signed-rank test demonstrated that 85% of comparisons were significantly different. The PAQs for ‘calm’, ‘eventful’, ‘pleasant’, ‘chaotic’, ‘monotonous’, and ‘uneventful’ corroborated with the null hypothesis, that is, they changed with the number of people in the scenario (Fig 3A–3F). The ‘annoying’ in the ‘Plaza’ for the LAB VR (Fig 3A), the ‘vibrant’ of all locations in the MCR online (Fig 3A–3C), and the same attribute at the ‘Park’ in the LAB VR (Fig 3E) were also significantly different with population densities. When relating to the ‘Plaza’, results corroborate with the strategic urban plan done in 2016 to improve Piccadilly Gardens (‘Plaza’) into a vibrant location [56]. These similar results may indicate that both experiment methods were equivalent, given recordings, methods, and locations were the same, but in different moments. That is, perceptions of calmness always changed with population density at the ‘Park’ as did perceptions of eventfulness, pleasantness, uneventfulness, chaotic, and monotonous changed at the pedestrian street (‘Street’). This observation points out that these attributes may be sound qualities to consider when studying similar locations.

In the ‘Plaza’, there was a constant water fountain sound. This sound could mask the background traffic noise, which can cause a positive sensation that could justify the same pleasant rating. This masking effect was also observed in the study related to environmental noise [57]. Similar results related to the ‘pleasant’ and ‘vibrant’ qualities of water features showed that three Naples waterfront sites had no differences among laboratory and online experiments [32]. This finding corroborates the concept of using water sound as a tool [58, 59] to support urban sound management and planning [9, 38].

When verifying the hypothesis (H_02_) regarding differences among urban locations in the same population density and experimental method, the Mann-Whitney test presented 63% and 58% significant differences for the MCR online and the LAB VR, respectively. The ‘calm’ PAQ was significantly different among four comparing sites for the MCR online (Fig 4A, 4B, 4D and 4E). Meanwhile, the LAB VR had five comparing sites (Fig 5B–5F) which corroborates with the null hypothesis. This tendency indicates that the ‘calm’ soundscape quality may be easier to assess since quiet areas are the opposite of noise pollution. However, there is a misconception of the definition of ‘calm’, which is easily confused with the term ‘quiet’. The ‘calm’ term represents pleasant and harmonic sound sources, while the ‘quiet’ term refers to the absence of sound sources. The calmness is more associated with silence, relaxation, and a tranquil area [60]. In addition, regarding the empty locations, resemblances among scores may be expected, given early hours may evoke similar perceptions. The tendency of similar results was unexpected for the comparison among the park and plaza (Fig 4F), given that different space functionalities may indicate different soundscape ‘characters’ as observed by Bento Coelho [38] and Siebein [53].

In both experiments, neutral responses, considered here as values around zero, were observed with 56% for the Wilcoxon signed-ranked test, and 54% and 44% for the Mann-Whitney test at the MCR online and LAB VR, respectively (Figs 3–5). Such behaviour might be related to neutral emotions which are also common in public opinion polls, because people avoid conflicting issues, especially when indifferent, and not used to the research topic or location [61, 62]. Furthermore, neutrality may be because of a lack of familiarity with location due to the absence of retrieved sound memory [63]. Since semantic memory consists of facts, concepts, data, general information, and knowledge [64], individuals’ opinions must be grounded in these elements to interpret and rate the sonic environment [65]. For example, in the Wilcoxon signed-rank test the busy condition, the ‘monotonous’ and ‘uneventful’ scales were around zero in the same compared locations in both methods (Fig 3). Meanwhile, in the Mann-Whiteney test, unexpected similarities were observed in the MCR online within half compared locations for the ‘monotonous’ scale with values over zero (Fig 4). Similar zero scores were observed in the location comparisons for the ‘chaotic’, ‘annoying’, and ‘eventful’ qualities in the ‘Plaza’ vs. ‘Park’ empty in both experimental methods (Figs 4 and 5).

Another possibility for the neutrality of responses may be due to the uniformity of soundscapes which gives an impression of ‘blended’ sounds. This sound could be denominated as a ‘blended urban soundscape’, common in big cities due to similar sound sources in different functioning landscapes, also identified by Schafer as a ‘lo-fi’ sound [40]. When the environment is excessively urbanised, where the population exceeds three million inhabitants, the sonic environment is somehow normalised, so that people do not identify differences among the diverse urban soundscapes. These urban sonic environments are dominant in traffic and human-made sounds, constantly present in the background, and natural sounds have become rare. These noises could cause neurological stress on the population, where they become anesthetised due to overwhelming urban sounds. As Le Van Quyen [66] recommended, urban citizens should practice a ‘mental detox’, which includes being in a quiet environment. Such a principle reinforces the importance of maintaining and preserving quiet areas. It is also important to notice that these ‘blended soundscapes’ should be avoided when designing urban sound zones, to give character [38, 53] and create diversity [67] within each site.

Another factor may be socio-cultural differences since 50% of participants from the MCR online were Brazilian Portuguese speakers. Some PAQ English words may not represent a common term in the Brazilian Portuguese language, as observed in Antunes et al. [68]. These inconsistencies in translations were also encountered in participating countries of the SATP group [14], as observed in the Indonesian study [15]. Therefore, further investigations should continue to consolidate the English terminology [4] so that translations can improve. However, even though there was a neutrality of perceived responses, the psychoacoustic indicators for the ‘Plaza’ busy scene showed higher values in loudness, sharpness, and tonality due to the sound source characteristics of the location. The most common sound sources in this location were the water sound from the fountain, children playing and shouting (sharpness, loudness, and tonality), tram circulation and sounds of tram brakes (sharpness and tonality), and babble sounds (loudness) [17, 69]. Most psychoacoustic indicators in the other locations and densities presented similar results, corroborating with the characteristics of the ‘blended’ soundscapes.

Limitations of this work consist of audio levels and different smartphone audio reproduction in the online experiment, as well as lack of familiarity with the study areas, ‘social desirability’ in which participants desire to please the researcher [70], and ‘experimenter effect’ where individuals need to use their critical thinking in a way they never had to do before [71]. Recommendations are to adjust audio levels to the field sound levels at the beginning of an online experiment [72]. In the case of smartphone use in the online experiments, it is also recommended to ask the participant to inform the brand of the device to verify the factory calibration of loudspeakers.

## 5. Conclusions

This work aimed to observe the PAQ results regarding differences among the two population densities for each location, and comparisons among locations for each experimental method. The study highlighted that there were significant results regarding the effect of population density and comparison among locations in the subjective responses. Still, the neutrality of results did not contribute to characterising the soundscape diversity in a megalopolis city. Meanwhile, the second hypothesis verified that the differences among locations within each experimental method demonstrated similar unexpected results. Such behaviour was discussed and could be related to the participants’ unfamiliarity with the location, and homogeneities of the urban sonic environment characterized here as ‘blended urban soundscapes’.

Based on the identified ‘blended soundscapes’, it is highlighted the importance of managing and planning the sonic environment by the clear delimitation of the acoustic zones in line with the functionality of the space. Furthermore, soundscape tools should be investigated to increase the diversity of sound sources, enhancing the sonic environment with elements such as masking, bio-phony, noise reduction, noise barriers, selection of urban materials, and sound art installations, among others.

Future works include evaluating other cities with lower population density to highlight the PAQs to avoid ‘blended’ soundscapes and enrich the sonic environment for VR experiments. Further neurologic evaluations must include more objective metrics in assessing cognitive responses to urban soundscapes and understanding how social-cultural differences are reflected in VR experiments. These VR findings can support urban design in a low-cost approach where urban planners can test different scenarios and interventions.

## Supporting information

S1 Data (XLSX)
