# Correction: Evaluating the perceived affective qualities of urban soundscapes through audiovisual experiments

**DOI:** 10.1371/journal.pone.0324650

**Published:** 2025-05-20

**Authors:** The PLOS One Staff

There are errors in the author affiliations. The correct affiliations are as follows: Maria Luiza de Ulhôa Carvalho^1,2*^, Margret Sibylle Engel^3,4^, Bruno M. Fazenda^2^, William J. Davies^2^ **1** Faculty of Visual Arts, Federal University of Goiás, Goiânia, Brazil, **2** Acoustics Research Centre, University of Salford, Manchester, United Kingdom, **3** Chair of Acoustics and Haptics, Technische Universität Dresden, Dresden, Germany, **4** Environmental Research & Innovation Centre, University of Salford, Manchester, United Kingdom.

The publisher apologizes for the error.

## References

[1] Carvalho MLdU, Engel MS, Fazenda BM, Davies WJ. Evaluating the perceived affective qualities of urban soundscapes through audiovisual experiments. PLoS One. 2024;19(9):e0306261. doi: 10.1371/journal.pone.0306261 39236001 PMC11376513

